# Ketogenic metabolic therapy in the remission of chronic major depressive disorder: a retrospective case study

**DOI:** 10.3389/fnut.2025.1549782

**Published:** 2025-02-27

**Authors:** Nicole Laurent, Erin L. Bellamy, Donika Hristova, Ally Houston

**Affiliations:** ^1^Family Renewal, Inc., Vancouver, WA, United States; ^2^School of Psychology, University of East London, London, United Kingdom; ^3^Transformation Evoked, Virginia, MN, United States; ^4^Department of Psychiatry, University of Oxford, Oxford, United Kingdom

**Keywords:** ketogenic diet, ketogenic metabolic therapy, major depressive disorder, psychiatric recovery, case report, metabolic psychiatry, MDD

## Abstract

**Background:**

There is limited evidence describing the use of ketogenic metabolic therapy (KMT), also known as a ketogenic diet (KD), to achieve full remission of treatment-resistant major depressive disorder (MDD) in real-world clinical settings. This case study examines a 47-year-old woman with lifelong treatment-resistant MDD who achieved complete remission of depressive symptoms and improved functioning through a ketogenic diet.

**Methods:**

The patient engaged in KMT with a 1.5:1 macronutrient ratio under the supervision of a treatment team consisting of a medical professional, psychotherapist, and ketogenic-informed nutrition professional through an online program that provided both individual and group support. Interventions included dietary modifications, micronutrient supplementation, and participation in a group coaching program. Outcomes were assessed using validated tools for symptom severity, including PHQ-9 for depression and GAD-7 for anxiety, at baseline, 2 months, and 4 months post-intervention. Qualitative data on patient experiences and functional improvements were also collected.

**Results:**

The patient achieved remission of MDD within 8 weeks of initiating KMT, with PHQ-9 scores decreasing from 25 (severe depression) at baseline to 0 at 2- and 4-month assessments. GAD-7 scores decreased from 3 (minimal anxiety) to 0 over the same period. Qualitative findings revealed significant improvements in emotional regulation, energy levels, and cognitive function.

**Conclusion:**

This case study demonstrates the potential of KMT as a non-pharmacological intervention for achieving full remission in treatment-resistant MDD. These findings suggest further research to evaluate feasibility, efficacy, and broader applicability in diverse clinical settings.

## 1 Introduction

Major depressive disorder (MDD) is a serious mental health condition characterized by a persistent low mood, loss of interest or pleasure, changes in weight or appetite, sleep disturbances, fatigue, feelings of worthlessness or guilt, difficulty concentrating, and recurring thoughts of death or suicide. To meet the diagnostic criteria, these symptoms need to have lasted for at least 2 weeks and cause significant problems in daily life (1). MDD affects a large number of people worldwide, with the number of new cases rising by 59%, increasing from 172.7 million in 1990 to 274.8 million in 2019 (2). Having repeated episodes of this disorder (3, 4) is a leading cause of disability, affecting both mental well-being and creating a substantial socioeconomic burden (2, 5).

Treatment for MDD often includes antidepressant medication, psychotherapy, or a combination of these approaches, with psychotherapy alone being reported to consistently outperform in lowering the risk of relapse (6, 7). A recent meta-analysis evaluating the effectiveness of psychotherapies for depression examined rates of response, remission, and deterioration compared to control conditions such as usual care, waitlist, and pill placebo. It found that psychotherapies were more effective than control conditions, but more than half of the patients did not respond, and only about one-third achieved remission (8). However, clinical research indicates that nearly 50% of people on antidepressants continue to experience significantly impaired quality of life, even if their symptoms improve (9). Many people struggle to tolerate the medications used for treating depression due to adverse drug reactions (ADRs) (10, 11). This can lead to significant non-adherence to medication regimens, with dropout rates ranging from 46 to 83% in patients who experience side effects such as somnolence, emotional numbing, and headaches, which contributes to their discontinuing treatment (10). Antidepressants, including tricyclic antidepressants and SSRIs, are linked to various metabolic changes. These include weight gain, cardiac alterations, diabetes, gastrointestinal disorders, cardiovascular alterations, constipation, and alterations in the intestinal flora can pose significant risks to patients (12). Although a 2024 systematic review and meta-analysis reported outcomes using antidepressants as “clinically satisfying” (13), these assessments may fail to account for the significant quality-of-life impairments and patient dissatisfaction documented in other studies that have found many individuals on antidepressant medications continue to have a poor quality of life, even when their symptoms seem to improve (14). This incongruent reporting indicates that standard treatment may not fully address patient-centered outcomes (15).

Researchers have observed impaired brain metabolism, including glucose metabolism, in key regions such as the prefrontal cortex and hippocampus in people with MDD (16–19). Despite the multifaceted nature of MDD, studies continue to emphasize the role of neurotransmitter dysfunction within broader neurobiological frameworks (20). Reports indicate neuroinflammation, characterized by insulin resistance and elevated inflammatory markers linked to symptom severity and treatment resistance (21–24), coupled with increased oxidative stress in depression. This oxidative stress, marked by diminished antioxidant defenses, contributes to neuronal damage and reduced neuroplasticity (25, 26).

Ketogenic metabolic therapy (KMT), often referred to as the ketogenic diet (KD), is a dietary approach characterized by low carbohydrate intake, moderate protein consumption, and high fat intake (27). A 2022 review suggested substantial overlap between the mechanisms of MDD and the potential mechanisms by which a ketogenic diet could affect MDD, including improvements in mitochondrial function, neurotransmitter balance, and reductions in inflammation (28). It has been shown that a ketogenic diet enhances mitochondrial function and reduces oxidative stress, which may improve neuronal function (29, 30). It has also been shown that ketone bodies can exert anti-inflammatory effects (31), which could lower elevated inflammatory markers associated with MDD (23).

A prior case series and a retrospective clinical analysis have previously investigated the use of KMT for treating MDD. The case series reported full remission of depression in three patients diagnosed with both MDD and generalized anxiety disorder following a 12-week KMT intervention (32). In a retrospective analysis, six out of 27 participants had a primary diagnosis of MDD, with their mean Hamilton Depression Rating Scale (HAM-D) score decreasing from 24.0 (severe depression) to 5.5, determined as within normal range (33). While the duration of the intervention ranged from six to 248 days across all diagnoses without specific data for MDD, the study noted that clinical response to the ketogenic diet typically occurred within 3 weeks.

A number of trials are currently investigating the efficacy of a KD in treatment-resistant depression and moderate-to-severe MDD, advancing understanding of its therapeutic applications. These include the Ketogenic Diet for Treatment-Resistant Depression: Dietary Interventions for Mental Health Study (DIME), Ketogenic Diet for Depression (KDEP), Ketogenic Diet for Microbiome Optimization and Overcoming Depression (KETO-MOOD), and Ketogenic Intervention in Depression (KIND) (34–37).

While these trials aim to establish the efficacy of a KD through large-scale investigations, individual case studies provide crucial insights into real-world applications and patient-centered outcomes. This case study explores the potential of KMT to address MDD by evaluating its impact on symptom remission and daily functioning.

## 2 Case presentation

### 2.1 Clinical background

A 47-year-old woman who experienced long-term depressive symptoms with a formal diagnosis of MDD. She had a long history of psychiatric illness, beginning with clinically significant depressive symptoms at the age of 13. She reported severe depressive symptoms, characterized by persistent low mood, extreme fatigue, and comorbid periods of anxiety experienced up to three times a week, during which she would cry uncontrollably and feel overwhelmed. She also reported great difficulty initiating daily tasks, described often being unable to wake up or get out of bed, and said she stayed in her pajamas for days at a time. Basic self-care, such as showering, was a struggle, and she described herself as “barely functioning.”

Over the ensuing 25 years, she had a consistent pattern of treatment-resistant MDD, during which time her depression became chronic and progressively more severe, marked by recurrent episodes of debilitating low mood and fatigue. She also reported chronic anxiety and described feeling emotionally trapped in a “very dark place.” Her lack of energy left her incapable of handling routine responsibilities, and she used alcohol four or more times per week to cope with emotional distress. Her symptoms worsened over time, culminating in what she reported as one of her most severe depressive episodes ever experienced.

The patient’s long treatment history involved a series of interventions, none of which provided lasting relief. She engaged in psychotherapy, which offered only limited benefits and failed to significantly improve her overall condition. She attempted duloxetine but her side effects led her prescriber to discontinue use. After this negative experience with side effects, she chose to avoid pharmaceutical treatments. As well as conventional treatments, she regularly took a whole-foods-based multivitamin that included plant-based sources of micronutrients, and she had adhered to a vegan diet for over 15 years, more recently adopting a Mediterranean diet, without improvement. During her time on the vegan diet, she developed iron deficiency anemia for which her doctor prescribed a supplement, but she did not experience improvements in her mood and energy levels.

### 2.2 Ketogenic metabolic therapy intervention strategy

The patient had a family member suffering from serious mental illness who had tried ketogenic metabolic therapy (KMT). The subject of our case study researched KMT and pursued treatment for herself. Her treatment team consisted of a medical doctor, a psychotherapist, and a ketogenic-informed nutritional professional, who initiated and monitored the diet using an online program that provided self-paced education, access to two live question-and-answer group sessions a week, and access to private consultations to review relevant blood work and adjust macros. She was unmedicated before KMT started and remained unmedicated throughout this intervention. Her baseline weight was 150lbs (68 kg) and BMI was 25.7. These remained stable and without change during the 14-week treatment.

Supplementation provided included a methylated B-complex, trace minerals (zinc, copper, manganese, chromium, molybdenum, boron, and vanadyl sulfate), vitamins D and K, and electrolytes in the form of sodium, magnesium, calcium, and potassium. She also took fish oil at a dose of 2,000 mg, providing 650 mg EPA and 450 mg DHA. She continued iron supplementation provided by her doctor throughout her ketogenic intervention.

Macronutrient tracking was initiated using Cronometer, which identified an average baseline carbohydrate consumption of 67 with a daily range between 58 and 87 g. Macronutrient ratios were set at a modified-ketogenic ratio of 1.5:1 (163 g fat, 79 g protein, 30 g net carbohydrate). The use of 30 g net carbohydrates was initially set to allow for more plant-based protein options, although over time average carbohydrate intake reduced to a consistent 20 g net or less. She followed a diet that included fish and shellfish, eggs, dairy, and processed plant-based protein sources such as vegan ‘steak’ and plant ‘chicken.’ Primary fat sources included avocados, olive oil, medium chain triglyceride (MCT) oil, heavy cream, nuts and nut butters, which included macadamia nuts and peanut butter. The diet was complemented with low-carbohydrate vegetables and small amounts of low-carbohydrate berries. The patient followed a two-meal-a-day plan as this was her established eating pattern.

Approximately 2 weeks into consistent 20 g net carbohydrate restriction, lab work revealed free carnitine at 20 μmol/*L. prior* studies have defined free carnitine values less than 25 μmol/L to be considered in the low range (38). Nonprescription acetyl-L-carnitine supplementation was added at 2,000 mg per day, divided into two doses with meals. The supplement contained no carbohydrates.

**Figure 1:**
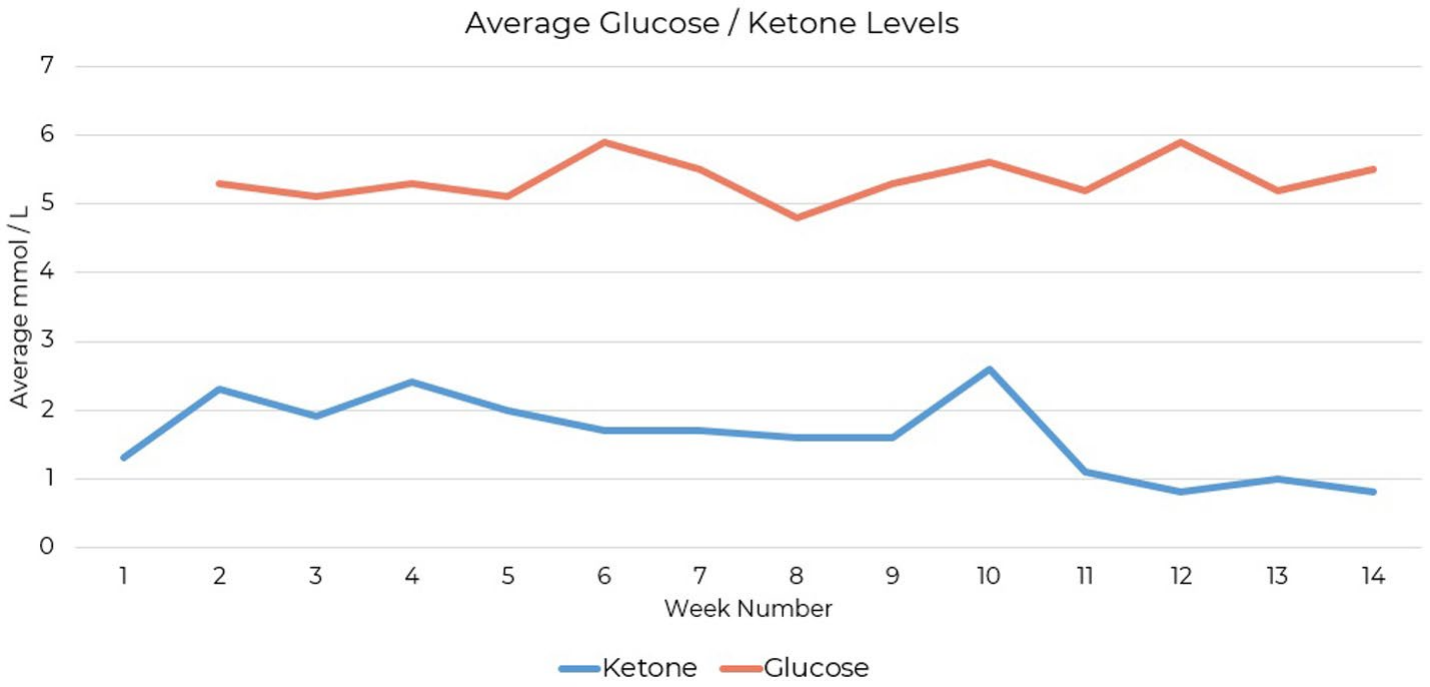
Blood β-hydroxybutyrate levels measured during ketogenic metabolic therapy using the Keto-Mojo® GK+ monitoring system. Initial ketosis was established at 1.1 mmol/L.

Testing compliance was 79% complete for daily ketone measures and 49% complete for daily glucose measures over the 14-week period. The patient tracked blood glucose and beta-hydroxybutyrate levels using the Keto-Mojo® GK+ blood glucose and β-ketone dual monitoring system, establishing initial ketosis at 1.1 mmol/L (Figure 1).

## 3 Evaluation of intervention outcomes

### 3.1 Quantitative analysis

**Figure 2:**
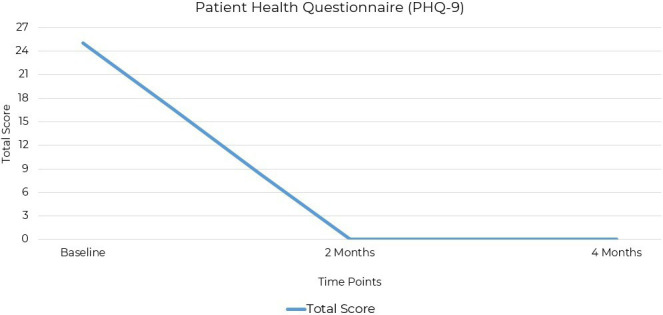
PHQ-9 scores at baseline, 2 months, and 4 months post-intervention. Depression severity decreased from 25 (severe) to 0 (remission) within 8 weeks of initiating ketogenic metabolic therapy.

The PHQ-9 (Patient Health Questionnaire-9) is a validated self-reporting tool for assessing depression severity. It consists of nine items based on DSM-5 criteria for MDD, scored from 0 to 3. The PHQ-9 has strong psychometric properties, including high reliability, validity, and sensitivity, and is used in clinical and research settings (39, 40). The patient’s depression symptoms, assessed using the PHQ-9, showed a total score of 25 at baseline out of a possible score of 27, indicating severe depression. Assessments at the two-month follow-up, and at the final assessment 2 months later, recorded scores of 0, with no depressive symptoms reported (Figure 2).

**Figure 3:**
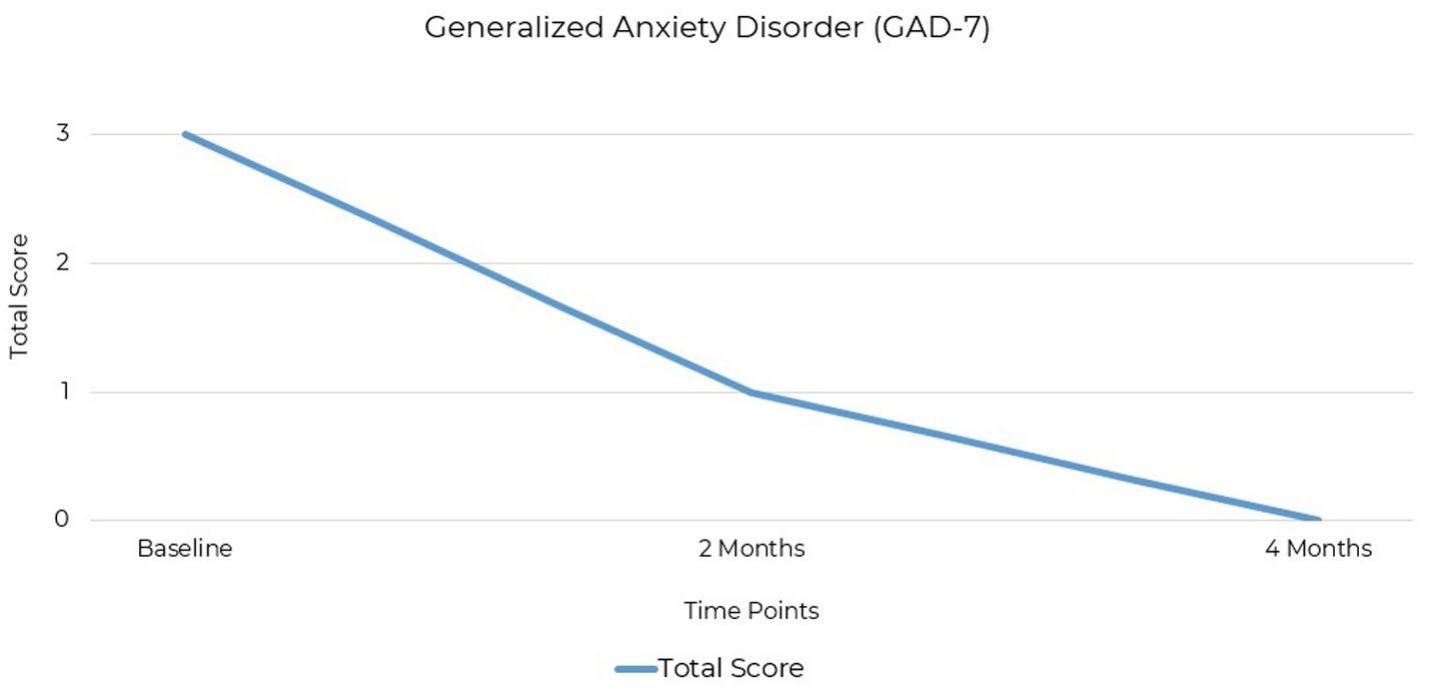
GAD-7 scores at baseline, 2 months, and 4 months post-intervention. Anxiety severity decreased from 3 (minimal) to 0 (remission) by the final assessment.

The GAD-7 (Generalized Anxiety Disorder-7) questionnaire is widely accepted and used in both clinical and research settings as a screening tool for anxiety (41, 42). It consists of seven items aligned with DSM-5 criteria for generalized anxiety disorder and uses a four-point scale from 0 to 3 to assess symptom severity. The GAD-7 is frequently used to identify and monitor anxiety symptoms and to evaluate treatment efficacy (43). The patient’s anxiety symptoms, assessed using the GAD-7, were initially within the minimal range with a baseline score of 3 out of a possible score of 21. At the two-month follow-up, the score was 1 and reached 0 at the final assessment 4 months after baseline (Figure 3).

Both these standardized mood assessments are well-established in clinical practice for quantifying the severity of depression and anxiety symptoms (Table 1). The significant change in scores from baseline to 2 months and the end of the treatment at 4 months demonstrated improvements in symptoms. Depression was the primary symptom, with no clinically significant comorbid anxiety.

**Table 1:** Overview of quantitative mental health assessment tools used in the MDD case study of KMT as a treatment.

Instrument name	What it measures	Consistency	Reliability	Validity	Cut-off scores
GAD-7 (41)	The severity and presence of generalized anxiety disorder symptoms	Strong internal consistency (α = 0.92)	Strong test–retest reliability (ICC = 0.83)	Strong construct validity	Anxiety severity: 0–4: Minimal 5–9: Mild 10–14: Moderate 15–21: Severe (39, 40, 49)
PHQ-9 (39, 40, 49)	The presence and severity of depression symptoms	Good internal consistency (α = 0.839)	Strong test–retest reliability (ICC = 0.84)	Strong convergent and construct validity	Depression severity: 1–4: None 5–9: Mild 10–14: Moderate 15–19: Moderately severe 20–27: Severe

### 3.2 Qualitative analysis

After starting the ketogenic diet, the patient reported remarkable improvements across multiple domains of functioning, including a marked increase in energy levels, which allowed her to more effectively manage parenting and household responsibilities, particularly in her ability to handle stressors and respond to external demands. After 2 months adherence to the diet, she reported a complete remission of MDD, including relief from the pervasive sadness and emotional dysregulation she had experienced in depressive episodes. She also reported perceiving he environment more positively. Observations of these improvements was reported to be collaborated by family members and other members of the treatment team. The patient also described self-perceived enhancements in cognitive processing that she said had led her to participate more meaningfully in her treatment plan. She described the impact of these changes, stating, “I’m going back to school to get a master’s in nutrition science; the program starts next spring! I feel that my brain is ready to learn new things and that I am able to start a new career.”

## 4 Discussion

The patient self-selected and pursued KMT with the specific goal of addressing her psychiatric symptoms rather than seeking it out for the more usual motivation of weight loss. The remission of her chronic MDD achieved through KMT demonstrates the potential for non-pharmacological interventions in clinical settings when individualized dietary modifications and structured support are provided.

This case study demonstrates the flexibility required in clinical practice, where treatment evaluations and protocols are tailored to meet individual patient needs. The timing of assessments, for example, was decided using clinical judgment that prioritized the patient’s specific circumstances rather than adhering to predetermined schedules. The use of a pescatarian dietary approach and targeted micronutrient supplementation further demonstrates the adaptability of KMT to accommodate patient preferences and nutritional needs.

The patient also used and benefited from a group coaching program designed and led by a single ketogenic-informed professional within a treatment team that consisted of a medical doctor and a psychotherapist. The role of accessible and targeted guidance in supporting adherence and achieving therapeutic outcomes with KMT could be clinically explored with different treatment practitioners and in different settings.

A prior case series and retrospective analysis reported remission of depression and improvements in mood stability with KMT within three to 16 weeks (32, 33). The outcomes of our case study, with remission achieved in 8 weeks, are consistent with these timelines, reflecting similar patterns of symptom improvement in MDD. Unlike previous studies, however, our case study involved an unmedicated patient, minimizing the potentially confounding effects of psychotropic medications commonly present in similar case reports.

This case study provides compelling evidence of the benefits of KMT for MDD, though we do acknowledge several limitations. First, a single case study lacks generalizability. Second, the patient benefited from a structured support system and personalized dietary modifications that may have contributed to her high level of adherence. This may be challenging to replicate in wider populations where support is limited or less personalized or limited. This is something that requires further investigation.

The inclusion of acetyl-L-carnitine supplementation presents a confounding factor in this case study as it has been shown to optimize mitochondrial function and provide neuroprotective effects (44). This may have contributed to the rapid response observed. Clinicians should continue to test carnitine levels where possible and future research should assess whether hypocarnitinemia contributes to the development or severity of mental health conditions as is seen in those with MDD and epilepsy (38, 45). This may provide useful information for clinicians using ketogenic therapy as a treatment for mental illness.

Future research should also prioritize clinical and randomized controlled trials to establish causality and efficacy across diverse populations with treatment-resistant depression. Research should explore optimal dietary formulations, including variations in macronutrient ratios, and supplementation strategies, that lead to clinically meaningful improvements.

Finally, there is a critical need for qualitative studies that explore patients’ experiences in implementing ketogenic therapy and its impact on daily life (46, 47). This observation aligns with reports that some patients identify as being in remission even when symptom-based measures indicate otherwise, suggesting that subjective experiences play an important role in understanding recovery (48). Understanding the subjective aspects of treatment, such as challenges, perceived benefits, and changes in quality of life, can provide invaluable insights into the feasibility and long-term effectiveness of ketogenic therapy for diverse populations suffering from MDD.

## 5 Conclusion

This case study reports remission of lifelong MDD achieved within 2 months of KMT, with clinical improvements in emotional regulation, energy levels, and overall daily functioning. These results were corroborated by standardized mood assessments and reports from the patient’s family and treatment team, indicating a promising area for further research.

Full recovery from MDD should include not only clinical assessments but also the patient’s psychosocial functioning and self-perception of remission. Residual symptoms and cognitive impairments, both objective and subjective, are crucial factors and should be specifically addressed in therapeutic strategies (48). This single case study addresses these factors by presenting quantitative and qualitative data demonstrating remission, corroborated by the patient’s self-reporting of remission.

## Data Availability

The original contributions presented in the study are included in the article/supplementary material, further inquiries can be directed to the corresponding author.

## References

[1] Diagnostic and Statistical Manual of Mental Disorders (2022). Psychiatry online. DSM Library. doi: 10.1176/appi.books.9780890425787 (Accessed November 4, 2024)

[2] Yan G Zhang Y Wang S Yan Y Liu M Tian M. Global, regional, and national temporal trend in burden of major depressive disorder from 1990 to 2019: an analysis of the global burden of disease study. Psychiatry Res. (2024) 337:115958. doi: 10.1016/j.psychres.2024.115958, PMID: 38772160

[3] Hasin DS Sarvet AL Meyers JL Saha TD Ruan WJ Stohl M. Epidemiology of adult DSM-5 major depressive disorder and its specifiers in the United States. JAMA Psychiatry. (2018) 75:336–46. doi: 10.1001/jamapsychiatry.2017.4602, PMID: 29450462 PMC5875313

[4] McIntyre RS Alsuwaidan M Baune BT Berk M Demyttenaere K Goldberg JF. Treatment-resistant depression: definition, prevalence, detection, management, and investigational interventions. World Psychiatry. (2023) 22:394–412. doi: 10.1002/wps.21120, PMID: 37713549 PMC10503923

[5] Greenberg P Chitnis A Louie D Suthoff E Chen S-Y Maitland J. The economic burden of adults with major depressive disorder in the United States (2019). Adv Ther. (2023) 40:4460–79. doi: 10.1007/s12325-023-02622-x, PMID: 37518849 PMC10499687

[6] Zainal NH. Is combined antidepressant medication (ADM) and psychotherapy better than either monotherapy at preventing suicide attempts and other psychiatric serious adverse events for depressed patients? A rare events meta-analysis. Psychol Med. (2024) 54:457–72. doi: 10.1017/S0033291723003306, PMID: 37964436

[7] Schefft C Guhn A Brakemeier E-L Sterzer P Köhler S. Efficacy of inpatient psychotherapy for major depressive disorder: a meta-analysis of controlled trials. Acta Psychiatr Scand. (2019) 139:322–35. doi: 10.1111/acps.12995, PMID: 30520019

[8] Cuijpers P Karyotaki E Weitz E Andersson G Hollon SD van Straten A. The effects of psychotherapies for major depression in adults on remission, recovery and improvement: a meta-analysis. J Affect Disord. (2014) 159:118–26. doi: 10.1016/j.jad.2014.02.026, PMID: 24679399

[9] Niarchou E Roberts L Naughton BD. What is the impact of antidepressant side effects on medication adherence among adult patients diagnosed with depressive disorder: a systematic review. J Psychopharmacol (Oxf). (2024) 38:127–36. doi: 10.1177/02698811231224171, PMID: 38344912 PMC10863360

[10] Read J Williams J. (2018). Adverse effects of antidepressants reported by a large Internatio…: Ingenta connect. Available at: https://www.ingentaconnect.com/content/ben/cds/2018/00000013/00000003/art00006 (Accessed November 8, 2024).

[11] Kearns B Cooper K Orr M Essat M Hamilton J Cantrell A. The incidence and costs of adverse events associated with antidepressants: results from a systematic review, network meta-analysis and multi-country economic model. Neuropsychiatr Dis Treat. (2022) 18:1133–43. doi: 10.2147/NDT.S356414, PMID: 35698594 PMC9188369

[12] Sepúlveda-Lizcano L Arenas-Villamizar VV Jaimes-Duarte EB García-Pacheco H Paredes CS Bermúdez V. Metabolic adverse effects of psychotropic drug therapy: a systematic review. Eur J Investig Health Psychol Educ. (2023) 13:1505–20. doi: 10.3390/ejihpe13080110, PMID: 37623307 PMC10453914

[13] Naher S Alramahi M Arida AK Arida A. A comparative efficacy of antidepressants in the treatment of major depressive disorder: a systematic review and meta-analysis. J Sociol Psychol Relig Stud. (2023) 5:40–69. doi: 10.53819/81018102t4218

[14] Paludan-Müller AS Sharma T Rasmussen K Gøtzsche PC. Extensive selective reporting of quality of life in clinical study reports and publications of placebo-controlled trials of antidepressants. Int J Risk Saf Med. (2021) 32:87–99. doi: 10.3233/JRS-200051, PMID: 33044196

[15] Wiesinger T Kremer S Bschor T Baethge C. Antidepressants and quality of life in patients with major depressive disorder – systematic review and meta-analysis of double-blind, placebo-controlled RCTs. Acta Psychiatr Scand. (2023) 147:545–60. doi: 10.1111/acps.13541, PMID: 36905396

[16] Su H Zuo C Zhang H Jiao F Zhang B Tang W. Regional cerebral metabolism alterations affect resting-state functional connectivity in major depressive disorder. Quant Imaging Med Surg. (2018) 8:910–24. doi: 10.21037/qims.2018.10.05, PMID: 30505720 PMC6218209

[17] Su L Cai Y Xu Y Dutt A Shi S Bramon E. Cerebral metabolism in major depressive disorder: a voxel-based meta-analysis of positron emission tomography studies. BMC Psychiatry. (2014) 14:321. doi: 10.1186/s12888-014-0321-9, PMID: 25407081 PMC4240898

[18] Pan LA Segreti AM Wrobleski J Shaw A Hyland K Hughes M. Metabolomic disorders: confirmed presence of potentially treatable abnormalities in patients with treatment refractory depression and suicidal behavior. Psychol Med. (2023) 53:6046–54. doi: 10.1017/S0033291722003233, PMID: 36330595 PMC10520591

[19] Penninx BWJH Lange SMM. Metabolic syndrome in psychiatric patients: overview, mechanisms, and implications. Dialogues Clin Neurosci. (2018) 20:63–73. doi: 10.31887/DCNS.2018.20.1/bpenninx, PMID: 29946213 PMC6016046

[20] Panigrahy A Prusty SK Panigrahi G. Neurobiological investigation of depression: unraveling the biological basis of a complex mental health challenge. Int J Pharm Qual Assur. (2023) 14:1287–95. doi: 10.25258/ijpqa.14.4.71

[21] Wu A Zhang J. Neuroinflammation, memory, and depression: new approaches to hippocampal neurogenesis. J Neuroinflammation. (2023) 20:283. doi: 10.1186/s12974-023-02964-x, PMID: 38012702 PMC10683283

[22] Réus GZ LuanaM M Quevedo J Carvalho AF. Major depressive disorder as a neuro-immune disorder: origin, mechanisms, and therapeutic opportunities. Neurosci Biobehav Rev. (2023) 155:105425. doi: 10.1016/j.neubiorev.2023.105425, PMID: 37852343

[23] Paganin W Signorini S. Inflammatory biomarkers in depression: scoping review. BJPsych Open. (2024) 10:e165. doi: 10.1192/bjo.2024.787, PMID: 39343996 PMC11536280

[24] Watson KT Simard JF Henderson VW Nutkiewicz L Lamers F Nasca C. Incident major depressive disorder predicted by three measures of insulin resistance: a Dutch cohort study. Am J Psychiatry. (2021) 178:914–20. doi: 10.1176/appi.ajp.2021.20101479, PMID: 34551583

[25] Sipahi H Mat AF Ozhan Y Aydin A. (2023). The interrelation between oxidative stress, Depression and Inflammation through the Kynurenine Pathway. Available at: https://www.eurekaselect.com/article/128411 (Accessed November 9, 2024). 10.2174/1568026623666221223111309 36567285

[26] Bhatt S Nagappa AN Patil CR. Role of oxidative stress in depression. Drug Discov Today. (2020) 25:1270–6. doi: 10.1016/j.drudis.2020.05.001, PMID: 32404275

[27] Zhu H Bi D Zhang Y Kong C Du J Wu X. Ketogenic diet for human diseases: the underlying mechanisms and potential for clinical implementations. Signal Transduct Target Ther. (2022) 7:11–21. doi: 10.1038/s41392-021-00831-w, PMID: 35034957 PMC8761750

[28] Shamshtein D Liwinski T. Ketogenic therapy for major depressive disorder: a review of neurobiological evidence. Recent Prog Nutr. (2022) 2:1–19. doi: 10.21926/rpn.2201003

[29] Greco T Glenn TC Hovda DA Prins ML. Ketogenic diet decreases oxidative stress and improves mitochondrial respiratory complex activity. J Cereb Blood Flow Metab. (2015) 36:1603–13. doi: 10.1177/0271678X15610584, PMID: 26661201 PMC5012517

[30] Drabińska N. Current perspective about the effect of a ketogenic diet on oxidative stress – a review. Pol J Food Nutr Sci. (2024) 74:92–105. doi: 10.31883/pjfns/185366, PMID: 33315315

[31] Puchalska P Crawford PA. Multi-dimensional roles of ketone bodies in fuel metabolism, signaling, and therapeutics. Cell Metab. (2017) 25:262–84. doi: 10.1016/j.cmet.2016.12.022, PMID: 28178565 PMC5313038

[32] Calabrese L Frase R Ghaloo M. Complete remission of depression and anxiety using a ketogenic diet: case series. Front Nutr. (2024) 11:1396685. doi: 10.3389/fnut.2024.1396685, PMID: 38887496 PMC11182043

[33] Danan A Westman EC Saslow LR Ede G. The ketogenic diet for refractory mental illness: a retrospective analysis of 31 inpatients. Front Psychol. (2022) 13:951376. doi: 10.3389/fpsyt.2022.951376, PMID: 35873236 PMC9299263

[34] Kakani G Alves TLIA Ata F Campos-Cuellar C Diaz DJA Gomes WF. The KDEP trial: protocol for a phase II, multicenter, open label, randomized controlled trial to evaluate the efficacy of ketogenic diet for symptomatic improvement of moderate to severe major depressive disorder. Princ Pract Clin Res. (2022) 8:77–83. doi: 10.21801/ppcrj.2022.83.10, PMID: 36787223

[35] Gao M Chang MK. (2024). A randomised controlled trial evaluating the efficacy and mechanisms of a ketogenic diet as an adjunctive treatment for people with treatment-resistant depression. [Clinical trial registration]. Available at: clinicaltrials.gov. https://clinicaltrials.gov/study/NCT06091163 (Accessed December 31, 2023). 10.1016/j.jpsychires.2024.04.023 38653031

[36] Liwinski T. (2023). KETO-MOOD: Ketogenic diet for microbiome optimization and overcoming depression. Available at: https://clinicaltrials.gov/study/NCT06105762 (Accessed July 19, 2024). 10.1159/000542979 PMC11844705 39701054

[37] Volek J. (2023). Study details | ketogenic intervention in depression. Available at: ClinicalTrials.gov. https://www.clinicaltrials.gov/study/NCT06080932?cond=depression&term=Ketogenic%20Diet&rank=2&limit=10 (Accessed July 19, 2024).

[38] Chu DY Ravelli MN Faltersack KM Woods AL Almane D Li Z. Hypocarnitinemia and its effect on seizure control in adult patients with intractable epilepsy on the modified Atkins diet. Front Nutr. (2024) 10:1304209. doi: 10.3389/fnut.2023.1304209, PMID: 38249600 PMC10796679

[39] Kroenke K Spitzer RL. The PHQ-9: a new depression diagnostic and severity measure. Psychiatr Ann. (2002) 32:509–15. doi: 10.3928/0048-5713-20020901-06

[40] Sun Y Kong Z Song Y Liu J Wang X. The validity and reliability of the PHQ-9 on screening of depression in neurology: a cross sectional study. BMC Psychiatry. (2022) 22:98. doi: 10.1186/s12888-021-03661-w, PMID: 35139810 PMC8827244

[41] Spitzer RL Kroenke K Williams JBW Löwe B. A brief measure for assessing generalized anxiety disorder: the GAD-7. Arch Intern Med. (2006) 166:1092–7. doi: 10.1001/archinte.166.10.1092, PMID: 16717171

[42] Omarsdottir HR Vésteinsdóttir V Asgeirsdottir RL Kristjansdottir H Thorsdottir F. Can GAD-7 be used reliably to capture anxiety?: approaching evaluation of item quality using IRT. Nord Psychol. (2024) 76:462–80. doi: 10.1080/19012276.2023.2279904

[43] Rutter LA Brown TA. Psychometric properties of the generalized anxiety disorder Scale-7 (GAD-7) in outpatients with anxiety and mood disorders. J Psychopathol Behav Assess. (2017) 39:140–6. doi: 10.1007/s10862-016-9571-9, PMID: 28260835 PMC5333929

[44] Wang W Pan D Liu Q Chen X Wang S. L-carnitine in the treatment of psychiatric and neurological manifestations: a systematic review. Nutrients. (2024) 16:1232. doi: 10.3390/nu16081232, PMID: 38674921 PMC11055039

[45] Nasca C Bigio B Lee FS Young SP Kautz MM Albright A. Acetyl-l-carnitine deficiency in patients with major depressive disorder. Proc Natl Acad Sci USA. (2018) 115:8627–32. doi: 10.1073/pnas.1801609115, PMID: 30061399 PMC6112703

[46] Laurent N. From theory to practice: challenges and rewards of implementing ketogenic metabolic therapy in mental health. Front Nutr. (2024) 11:1331181. doi: 10.3389/fnut.2024.1331181, PMID: 38389794 PMC10881829

[47] Bellamy EL Hadjiefthyvoulou F Walsh J Brown J Turner J. Understanding the experiences of ketogenic metabolic therapy for people living with varying levels of depressive symptoms: a thematic analysis. Front Nutr. (2024) 11:1397546. doi: 10.3389/fnut.2024.1397546, PMID: 38903620 PMC11188922

[48] Vicent-Gil M Serra-Blasco M Navarra-Ventura G Trujols J Balanzá-Martínez V Mj P. In pursuit of full recovery in major depressive disorder. Eur Arch Psychiatry Clin Neurosci. (2023) 273:1095–104. doi: 10.1007/s00406-022-01487-5, PMID: 36085532

[49] Kroenke K Spitzer RL Williams JB. The PHQ-9: validity of a brief depression severity measure. J Gen Intern Med. (2001) 16:606–13. doi: 10.1046/j.1525-1497.2001.016009606.x, PMID: 11556941 PMC1495268

